# Pro‐Aging: A New Approach to Beauty and Aesthetic Medicine

**DOI:** 10.1111/jocd.70277

**Published:** 2025-06-04

**Authors:** Stefano Fabbri, Mariachiara Fabbri

**Affiliations:** ^1^ Dentist and Aesthetic Medicine Practitioner Freelance Professional at Phimedical Pesaro Italy; ^2^ Plastic Surgery Resident, Residency Program in Plastic Surgery Università Cattolica del “Sacro Cuore” Rome Italy

**Keywords:** aesthetic medicine, aging face, anti‐aging

Beauty has always transcended time and culture, adapting to changing technologies, trends, and collective sensibilities. As these shifts continue, it is vital to reflect on our evolving relationship with beauty and how it shapes our identity in modern society.

The body is not merely an external shell; it is a vessel of lived experiences—joys, sorrows, and triumphs—that define who we are. As we age, physical changes can create a disconnect between how we feel internally and how we are perceived externally. This gap often leads to a sense of disorientation, where the reflection in the mirror no longer aligns with our self‐perception. In this tension between the past and future, there is a risk of losing sight of the present, the moment when our true essence can find its most authentic expression.

In an age where technology accelerates our lives and social media fosters idealized images of eternal youth, many are driven to align with these standards. This often leads to a struggle with aging and the mirror's reflection, which is shaped by time's inevitable passage. However, it is crucial to remember that our physical selves are not separate from our experiences—they embody the unique, irreplaceable story of a life lived fully.

This reflection leads us beyond the concept of Anti‐Aging, toward an alternative philosophy of Pro‐Aging, which emphasizes embracing aging as an integral part of our identity. Rather than striving to reverse the natural aging process, Pro‐Aging promotes accepting time's effects while enhancing and valuing our present selves. This shift requires a more mindful approach to beauty, one that celebrates the wisdom and authenticity that come with age.

Anti‐aging aesthetic medicine traditionally focuses on reversing physical signs of aging, often through chronologic or photo‐aging treatments aimed at restoring volume or mitigating sun damage [1, 2, 3]. While effective, these approaches often push an idealized, uniform standard of beauty that can create unrealistic expectations and overlook the individuality of aging. By contrast, Pro‐Aging recognizes that aging is not something to combat or erase, but a process to live with and cherish.

The Pro‐Aging approach in aesthetic medicine aims to enhance natural beauty by addressing the signs of aging in a conservative, personalized manner. Treatments such as microneedling [4], superficial or medium‐depth peels, and deep peels [5] work to stimulate the regeneration of collagen, elastin, and hyaluronic acid, leading to revitalized, youthful skin [6]. Botulinum toxin [7] is also used to smooth wrinkles caused by muscle contractions, preserving facial expressiveness and non‐verbal communication, which are vital to personal interactions, rather than eliminating all signs of age. The aim is not to become a caricature of oneself, but the best version possible. Sometimes, these conservative techniques must be complemented with fillers [8]. However, it is crucial to use them sparingly to soften and harmonize facial features without distorting them. For instance, plump lips are acceptable, provided they respect the right ratio between vermilion and skin, avoiding the “duck lip” or unnatural effects.

In contrast, the Anti‐Aging approach primarily aims to stop or even erase the signs of aging features that often reflect our identity and life experience. In this view, botulinum toxin is used more aggressively, not merely to smooth but to eliminate wrinkles by blocking muscle contractions, often through more frequent treatments. Fillers tend to be used more extensively as well, with the goal of restoring a youthful appearance. This may compromise natural facial harmony without addressing or preventing the deeper causes of aging.

The key difference lies in both the philosophy and the sequence of intervention: the Pro‐Aging approach starts by treating the skin and underlying structures, gradually restoring each layer, and only at the end may introduce volume to gently refresh the appearance. Volume, in this case, is a complement, not the central goal. By contrast, the Anti‐Aging approach begins by filling wrinkles and depressed areas and nearly paralyzing facial muscles, with skin quality being considered, if at all, only at a later stage. Over the years, the anti‐aging perspective has developed a kind of aesthetic medicine focused on reconditioning facial volumes, atrophied or affected by gravitational ptosis, often adhering to a stereotyped, uniform standard of beauty. This model treats aging as a defect to be corrected, rather than a natural stage of life to be lived with pride. But why strive to become younger at all costs when we can simply make the most of our current age? Why attempt to stop the clock when time represents our memory and the foundation of who we are today? This realization has sparked the Pro‐Aging revolution. Pro‐Aging emphasizes prevention without neglecting the option to mask small imperfections but, unlike anti‐aging, it favors a gentle approach that softens rather than eliminates them entirely.

The goal of Pro‐Aging is not to stop time, but to embrace it. It is a recognition that beauty is not defined by perfection, but by how we present the best version of ourselves at every stage of life. This philosophy respects the natural passage of time and encourages us to live fully in the present, rather than dwelling on the past or striving for an unattainable future.

Approaching beauty through a Pro‐Aging lens means preventing aging, while approaching beauty through an Anti‐Aging lens means intervening once wrinkles have become entrenched. In other words: pro‐Aging is the freedom to “be” rather than “appear,” the freedom to rethink one's face and body and recognize oneself in the best version. It is an awareness of the present without excessive nostalgia for the past. From the patient's perspective, the Pro‐Aging approach offers several advantages: it promotes self‐acceptance, reduces the pressure to conform to unrealistic standards, and minimizes the risk of overcorrection or unnatural results. It also encourages earlier, gentler interventions that support skin health and long‐term well‐being, rather than relying on aggressive procedures once damage has already occurred. This often results in more sustainable outcomes and a stronger alignment between appearance and identity.

In conclusion, Pro‐Aging represents a shift in how we view and practice aesthetic medicine. Rather than focusing on stopping the clock or achieving an idealized standard of youth, it advocates for a celebration of natural beauty and an active participation in life at any age. This philosophy, encapsulated by the idea of becoming a “Perennial,” offers a more balanced and mindful approach to beauty and aging.

## Conflicts of Interest

The authors declare no conflicts of interest.

## Data Availability

Data sharing not applicable to this article as no datasets were generated or analysed during the current study.
